# The association between maternal blood pressures and offspring size at birth in Southeast Asian women

**DOI:** 10.1186/s12884-014-0403-1

**Published:** 2014-12-02

**Authors:** Wai-Yee Lim, Yung-Seng Lee, Chuen-Seng Tan, Kenneth Kwek, Yap-Seng Chong, Peter D Gluckman, Keith M Godfrey, Seang-Mei Saw, An Pan

**Affiliations:** Saw Swee Hock School of Public Health, National University of Singapore, Block MD1, #11-01E, 12 Science Drive 2, Singapore, 117549 Republic of Singapore; KK Women’s and Children’s Hospital, 100 Bukit Timah Road, Singapore, 229899 Singapore; Department of Paediatrics, Yong Loo Lin School of Medicine, National University of Singapore and National University Health System, 1E Kent Ridge Road, NUHS Tower Block Level 12, Singapore, 119228 Singapore; Singapore Institute for Clinical Sciences, A*STAR, Brenner Centre for Molecular Medicine, 30 Medical Drive, Singapore, 117609 Singapore; Yong Loo Lin School of Medicine, National University of Singapore, 10 Medical Drive (MD6), Singapore, 117597 Singapore; Department of Obstetrics and Gynaecology, Yong Loo Lin School of Medicine, National University of Singapore and National University Health System, 1E Kent Ridge Road, NUHS Tower Block Level 12, Singapore, 119228 Singapore; Liggins Institute, The University of Auckland, Private Bag 92019, Victoria Street West, Auckland, 1142 New Zealand; MRC Lifecourse Epidemiology Unit (University of Southampton), Southampton General Hospital, Mailpoint 95, Southampton, SO16 6YD UK; NIHR Southampton Biomedical Research Centre, University of Southampton and University Hospital Southampton NHS Foundation Trust, Southampton, SO16 6YD UK

**Keywords:** Pregnancy, Blood pressures, Obesity, Birth weight

## Abstract

**Background:**

Maternal blood pressures in pregnancy is an important determinant of offspring size at birth. However, the relationship between maternal blood pressures and offspring’s size at birth is not consistent and may vary between ethnic groups. We examined the relationship between maternal peripheral and central blood pressures and offspring size at birth in an Asian multi-ethnic cohort, and effect modifications by maternal ethnicity and obesity.

**Methods:**

We used data from 713 participants in the Growing Up in Singapore Towards Healthy Outcomes study consisting of pregnant Chinese, Malay and Indian women recruited from two tertiary hospitals between 2009 to 2010. Peripheral systolic and diastolic blood pressures (SBP and DBP), and central SBP and pulse pressure (PP) were measured around 27 weeks of gestation. Biometric parameters at birth were collected from medical records.

**Results:**

After adjusting for maternal and fetal covariates, each 1-SD increase (10.0 mmHg) in central SBP was inversely associated with birth weight (−40.52 g; 95% confidence interval (CI) -70.66 to −10.37), birth length (−0.19 cm; −0.36 to −0.03), head circumference (−0.12 cm; −0.23 to −0.02) and placental weight (−11.16 g; −20.85 to −1.47). A one-SD (11.1 mmHg) increase in peripheral SBP was also associated with lower birth weight (−35.56 g; −66.57 to −4.54). The inverse relations between other blood pressure measures and offspring size at birth were observed but not statistically significant. Higher peripheral SBP and DBP and central SBP were associated with increased odds of low birth weight (defined as weight <2500 g) and small for gestational age (defined as <10^th^ percentile for gestational age adjusted birth weight). Maternal adiposity modified these associations, with stronger inverse associations in normal weight women. No significant interactions were found with ethnicity.

**Conclusions:**

Higher second-trimester peripheral and central systolic pressures were associated with smaller offspring size at birth, particularly in normal weight women. Findings from this study reinforces the clinical relevance of antenatal blood pressure monitoring.

**Electronic supplementary material:**

The online version of this article (doi:10.1186/s12884-014-0403-1) contains supplementary material, which is available to authorized users.

## Background

Birth weight is an important measure of intra-uterine growth. Various maternal and fetal factors are known to influence size at birth [1,2]. Amongst these factors, maternal blood pressure has been considered as an important determinant. Various epidemiological studies have suggested that maternal hypertension is associated with an increased risk of lower birth weight [3,4]. Reduced utero-placental function has been suggested as one possible mechanism because this has been found to occur in women with concurrent pre-eclampsia and fetal growth restriction [2].

Several studies have investigated the associations between offspring’s birth weight and maternal peripheral [3,5-17] and central blood pressures [9,13,14], with inverse relations reported in most studies [3,5-9,13-17], but not all [10-12]. Some studies have also suggested that the relation between maternal central blood pressures and size at birth may be more pronounced than peripheral blood pressures [13,14]. However, very few studies have examined both peripheral and central blood pressures and the sample sizes were small in previous studies [9,13,14].

There is evidence that the relation between maternal blood pressures and offspring’s birth weight were found to be stronger in Asian Indians than white or black women [15], and was more evident in normal weight women than obese women [6]. As the incidence of small for gestational age in Southeast Asian women is one of the highest in the world [18], examining inter-ethnic variation may enable specific and appropriate public health interventions.

Therefore, we aimed to simultaneously examine both maternal peripheral and central blood pressures in relation to size at birth, and to explore the possible effect modification by maternal ethnicity or adiposity in pregnancy in a Southeast Asian birth cohort of pregnant Chinese, Malay and Indian women.

## Methods

**Figure 1:**
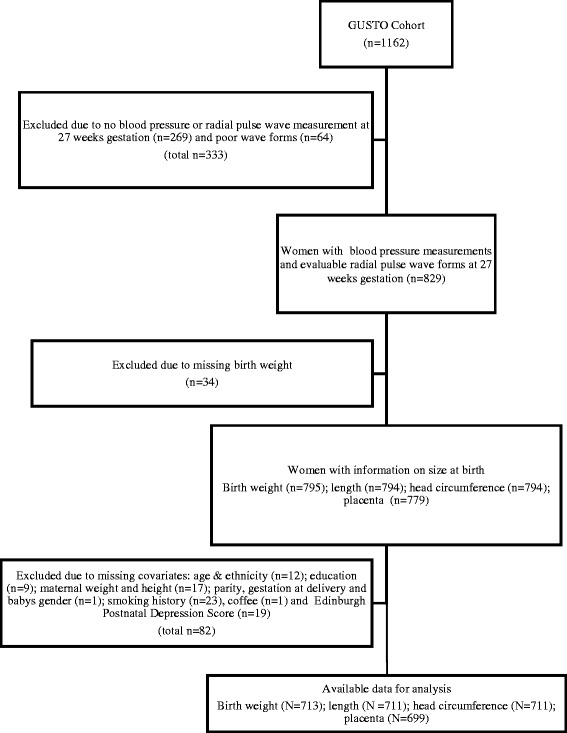
**Flow chart of the GUSTO study sample selected for analysis.**

The present study sample was drawn from the **G**rowing **U**p in **S**ingapore **T**owards Healthy **O**utcomes (GUSTO) study, a prospective early life cohort study comprising Chinese, Indian and Malay women [19]. Between 2009 and 2010, a total of 1162 pregnant women without type 1 diabetes or using chemotherapy or psychotropic drugs were recruited from two tertiary hospitals in Singapore. We excluded 333 women (28.6%) women who did not attend the blood pressure measurements or had incomplete recordings around 27 weeks, and 116 women who had incomplete demographic and pregnancy information, leaving a total of 713 women for the current analysis (Figure 1). Women who were excluded from the analysis had similar demographic characteristics compared with those who were included, although they had shorter gestation duration and smaller offsprings (Additional file 1: Table S1). The GUSTO study was approved by the SingHealth Centralized Institutional Review Board (CIRB Ref: 2009/280/D) and National Health Group Domain Specific Review Board (DSRB Ref: 09/021), and all participants have given informed consents.

At their enrolment visit prior to 14 weeks gestation, study participants were interviewed for baseline information on age, ethnicity, educational level, pre-pregnancy body weight, smoking history, coffee consumption and number of previous live-births. They were followed up at mid-pregnancy [median gestation of 27 weeks (interquartile range 26 to 29 weeks)] to measure maternal height and weight, as well as blood pressures using standard protocols [20]. Depression status (defined as an overall score of 13 or greater from the self administered Edinburgh Postnatal Depression Scale) and gestational diabetes (defined as fasting plasma glucose ≥7.0 mmol/L or 2-hour glucose ≥11.1 mmol/L) [21] was also examined during this visit. Maternal body mass index (BMI) before pregnancy and GUSTO mid-pregnancy follow-up visit were calculated as weight in kg divided by the square of height in meter, and categorised as normal weight for BMI <25.0 kg/m^2^, overweight as 25.0 to 29.9 kg/m^2^ and obese as ≥30.0 kg/m^2^ according to the WHO international classification [22]. Rate of weight gain was calculated as the weight difference before pregnancy and at GUSTO mid-pregnancy follow-up visit in kg divided by the length of gestation during mid-pregnancy in weeks.

Study participants were rested for at least 10 minutes prior to the first blood pressure measurement. Peripheral systolic and diastolic blood pressures (SBP and DBP) were taken on the right brachial artery at the level of the heart. Using an oscillometric device (MC3100, HealthSTATS International Pte Ltd, Singapore), three blood pressure readings were taken consecutively at 30 to 60 seconds apart to obtain the average reading of SBP and DBP. The A-pulse tonometer (BPro®, HealthSTATS International Pte Ltd, Singapore) was then applied on the radial artery of the same arm for continuous sampling of radial artery waveforms for at least 60 seconds. Central SBP was estimated from the calibrated radial artery waveforms [20], and central pulse pressure (PP) was calculated as the difference between central SBP and peripheral DBP [23].

Information on offspring size at birth (weight, length, head circumference and placental weight) were extracted from medical records, which were measured by midwives according to standard hospital protocols. Gestational age adjusted standard deviation (SD) scores for birth weight, length, head circumference and placental weight were constructed for the GUSTO cohort. The binary outcome of low birth weight was defined as weight at birth <2500 g, and small for gestational age was defined as those who were below the 10^th^ percentile for gestational age adjusted birth weight.

Blood pressure values were converted into SD scores, whereby per 1-SD increase in peripheral SBP and DBP was equivalent to 11.1 and 8.3 mmHg, respectively, and central SBP and PP to 10.0 and 6.5 mmHg, respectively. Maternal characteristics were compared across blood pressures using analysis of variance. The relationship between blood pressures and size at birth were examined using multiple linear and logistic regression models for continuous and binary birth size outcomes, respectively. All analyses were adjusted for baby’s sex, gestation age at delivery in weeks, maternal age, ethnicity, education, parity, smoking history, height, BMI around 27 weeks gestation, coffee consumption and depression. No adjustment was made for chronic hypertension as there were only 13 (1.8%) women with this condition.

We further evaluated the potential effect modification by maternal ethnicity (Chinese, Indian, or Malay) and BMI categories (normal, overweight, or obese). Multiplicative interaction terms with blood pressures as continuous variable and ethnicity or BMI as a categorical variable were added to the final model, and the likelihood ratio test was used to evaluate significance. We also reported the results stratified by maternal ethnicity (Chinese, Indian, or Malay) and BMI category (normal weight, overweight, or obese).

We performed a series of sensitivity analyses to test the robustness of our results: (1) we additionally adjusted for gestational diabetes (n = 663 due to 50 missing data for gestational diabetes status); (2) we examined a subgroup of 705 women within normal range of blood pressures (peripheral SBP and DBP below 140 and 90 mmHg, respectively); (3) we repeated our analysis using the imputed data (20 sets) for the missing information on blood pressures (imputed based on maternal age, ethnicity, education, parity, gestational diabetes, height and BMI around 27 weeks of gestation, and the respective outcome variable); (4) we adjusted for pre-pregnancy BMI and rate of weight gain instead of BMI around 27 weeks of gestation (n = 678 due to 35 missing data for pre-pregnancy weight); (5) we used tertiles of BMI around 27 weeks gestation instead of the WHO classification to test for interaction between maternal adiposity and blood pressures; (6) lastly, we examined gestational age adjusted size at birth measures as the outcomes instead of actual values to better account for the effect of gestational duration on size at birth. All analyses were performed using Stata version 11.2 (Statacorp, College Station, Texas), with statistical significance at two-sided p value less than 0.05.

## Results

Of the 713 women studied, 339 (55.9%) were Chinese, 196 (27.5%) were Indians and 118 (16.6%) were Malays. The mean age at enrolment was 30.5 (SD = 5.1) years. At the GUSTO study follow-up, 17.5% were obese at around 27 weeks of gestation (Tables 1 and Additional file 1: Table S1). Mean (SD) values for offspring’s birth weight, length, head circumference and placental weight were 3113.5 (435.0) g, 48.7 (2.2) cm, 33.4 (1.4) cm, and 585.3 (118.9) g, respectively. Women of Malay ethnicity, lower education levels and higher BMI categories were more likely to have higher peripheral and central blood pressures (p *<* 0.01, Table 1).

**Table 1:** **Distribution of maternal blood pressures by maternal characteristics** ^*^

**Maternal characteristics**	**No (%)**	**Peripheral systolic blood pressure (mmHg)**		**Peripheral diastolic blood pressure (mmHg)**		**Central systolic blood pressure (mmHg)**		**Central pulse pressure (mmHg)**	
		**Mean (SD)**	***P***	**Mean (SD)**	***P***	**Mean (SD)**	***P***	**Mean (SD)**	***P***
Age at booking (years)			0.54		0.26		0.62		0.36
1st quartile (18–26)	162 (22.7)	110.2 (12.0)		66.5 (8.7)		96.5 (10.6)		30.0 (6.9)	
2nd quartile (27–29)	148 (20.8)	109.6 (10.5)		67.1 (8.4)		96.3 (9.6)		29.3 (5.7)	
3rd quartile (30–33)	204 (28.6)	108.7 (11.0)		65.8 (7.8)		96.4 (9.8)		30.5 (6.7)	
4th quartile (34–46)	199 (27.9)	108.9 (10.7)		67.4 (7.9)		97.5 (9.8)		30.1 (6.4)	
Race			0.001				0.005		0.34
Chinese	399 (55.9)	108.4 (10.9)		66.3 (8.2)	0.004	95.9 (9.9)		29.7 (6.4)	
Indian	118 (16.6)	108.3 (10.8)		65.4 (7.9)		95.9 (9.3)		30.4 (7.1)	
Malay	196 (27.5)	111.8 (11.2)		68.2 (8.3)		98.7 (10.2)		30.4 (6.3)	
Education			0.005				0.001		0.84
Primary-Secondary	229 (32.1)	109.7 (11.5)		67.0 (8.4)	<0.001	97.2 (10.4)		30.2 (6.5)	
Post-Secondary	245 (34.4)	110.7 (11.4)		67.9 (8.2)		98.0 (10.0)		30.1 (6.7)	
Tertiary	239 (33.5)	107.5 (10.2)		64.9 (7.8)		94.8 (9.2)		29.8 (6.3)	
Smoking Status			0.01		0.29		0.14		0.34
Never smoker	618 (86.7)	108.9 (10.9)		66.4 (8.4)		96.5 (9.8)		29.9 (6.4)	
Ever smoker	95 (13.3)	111.9 (11.7)		67.5 (8.5)		98.1 (11.0)		30.6 (6.8)	
Coffee Consumption			0.80		0.45		0.39		0.73
No	369 (51.8)	109.2 (10.9)		66.5 (8.4)		96.4 (9.9)		29.9 (6.8)	
Yes	344 (48.2)	109.4 (11.2)		66.9 (8.0)		97.0 (9.9)		30.1 (6.2)	
Parity			0.47		0.72		0.26		0.17
Nulliparous	311 (43.6)	109.1 (11.0)		66.6 (7.9)		96.2 (9.6)		29.6 (6.7)	
Primiparous	246 (34.5)	108.9 (11.3)		66.4 (8.3)		96.6 (10.2)		30.2 (6.5)	
Multiparous	156 (21.9)	110.3 (10.7)		67.1 (8.5)		97.8 (10.2)		30.7 (6.0)	
Gestational Diabetes^**^			0.10		0.02		0.02		0.40
No	540 (81.4)	109.1 (10.7)		66.4 (7.9)		96.3 (9.6)		29.9 (6.4)	
Yes	123 (18.6)	110.9 (11.6)		68.2 (8.6)		98.7 (10.7)		30.5 (6.4)	
Pre-pregnancy BMI (kg/m^2^)^***^			<0.001		<0.001		<0.001		0.29
*<*25.0	518 (76.4)	107.0 (10.3)		65.1 (7.6 )		94.7 (9.4)		29.7 (6.5 )	
25.0-29.9	113 (16.7)	114 (9.7)		70.9 (8.3)		101.3 (8.8)		30.4 (6.4)	
≥30.0	47 (6.9)	119.4 (10.8)		73.4 (7.2)		104.3 (8.9)		30.9 (6.7)	
Rate of weight gain at 27 weeks (kg/week)^***^			0.005		0.004		0.006		0.80
1^st^ tertile (−0.42 – 0.25)	227 (33.5)	107.8 (11.0)		65.9 (8.6)		95.7 (10.1)		29.8 (6.6)	
2^nd^ tertile (0.26 – 0.37)	218 (32.2)	108.4 (10.2)		65.8 (8.1)		95.6 (9.1)		29.8 (6.6)	
3^rd^ tertile (0.38 – 1.38)	233 (34.5)	110.9 (11.2)		68.1 (7.7)		98.2 (10.0)		30.1 (6.3)	
Second trimester BMI (kg/m^2^)			<0.001		<0.001		<0.001		<0.001
*<*25.0	329 (46.2)	104.7 (9.9)		63.9 (7.7)		92.9 (9.3)		29.0 (6.2)	
25.0-29.9	259 (36.3)	110.9 (9.8)		67.3 (7.4)		98.2 (8.7)		30.8 (6.5)	
≥30.0	125 (17.5)	117.9 (10.6)		72.8 (7.4)		103.8 (9.4)		31.0 (6.7)	
Depression			0.99		0.71		0.46		0.52
Not depressed	631 (88.5)	109.3 (11.2)		66.7 (8.1)		96.8 (10.1)		30.1 (6.5)	
Depressed	82 (11.5)	109.3 (10.1)		66.4 (9.0)		95.9 (8.9)		29.6 (6.1)	

SD, standard deviation; BMI, body mass index.

^*^Data are represented as n (%) or mean (SD) where appropriate. *P* values were derived from analysis of variance.

^**^There were 50 women with missing values for gestational diabetes.

^***^There were 35 women with missing values for pre-pregnancy BMI and rate of weight gain.

After adjusting for maternal and fetal covariates, central SBP was inversely associated with all birth measures, and peripheral SBP was inversely associated with birth weight (all p *<* 0.05; Table 2). For example, each 1-SD increase (10.0 mmHg) in central SBP was inversely associated with birth weight (−40.52 g; 95% confidence interval [CI] -70.66 to −10.37), birth length (−0.19 cm; −0.36 to −0.03), head circumference (−0.12 cm; −0.23 to −0.02) and placental weight (−11.16 cm; −20.85 to −1.47). One-SD (11.1 mmHg) increase in peripheral SBP was also associated with 35.56 g lower birth weight (95% CI −66.57 to −4.54). The relations between other blood pressure measures and offspring size at birth were in the same direction but not statistically significant. Results were also not materially different in various sensitivity analyses (Additional file 1: Table S2).

**Table 2:** **Associations between blood pressures (per 1-SD increase) and size at birth** ^*^

**Measures of size at birth**	**N**	**Peripheral systolic blood pressure (1 SD = 11.1 mmHg)**	**Peripheral diastolic blood pressure (1 SD = 8.3 mmHg)**	**Central systolic blood pressure (1 SD = 10.0 mmHg)**	**Central pulse pressure (1 SD = 6.5 mmHg)**
		**ß (95% CI)**	**ß (95% CI)**	**ß (95% CI)**	**ß (95% CI)**
Weight (g)	713	−35.56 (−66.57 to −4.54)	−25.13 (−55.36 to 5.09)	−40.52 (−70.66 to −10.37)	−24.10 (−51.24 to 3.03)
Length (cm)^**^	711	−0.16 (−0.32 to 0.01)	−0.10 (−0.27 to 0.06)	−0.19 (−0.36 to −0.03)	−0.14 (−0.28 to 0.01)
Head circumference (cm)^**^	711	−0.09 (−0.19 to 0.02)	−0.08 (−0.18 to 0.02)	−0.12 (−0.23 to −0.02)	−0.07 (−0.16 to 0.02)
Placental weight (g)^***^	699	−8.78 (−18.74 to 1.19)	−6.94 (−16.63 to 2.76)	−11.16 (−20.85 to −1.47)	−6.44 (−15.04 to 2.16)

SD, standard deviation; CI, confidence interval.

^*^Multiple linear regression models were used with adjustment for baby’s sex, gestation at delivery, maternal age, ethnicity, education, parity, smoking history, height, BMI at 27 weeks gestation, coffee consumption and depression.

^**^There were 2 women with missing information on length and head circumference.

^***^There were 14 women with missing information on placental weight.

We found no significant interactions between blood pressures and ethnicity in relation to size at birth (Table 3). Stratified results for different ethnic groups showed that the associations between blood pressures (peripheral SBP, DBP and central SBP) and birth weight were significant in Chinese women only, but not significant in Malay or Indian women. However, the 95% CIs were large and tended to overlap among the three ethnic groups. We detected significant interactions between blood pressures and maternal BMI categories in relation to offspring’s birth weight, length and head circumference, with stronger associations in normal weight women rather than overweight/obese women (Table 4). Similar interactions were observed when tertiles of maternal BMI around 27 weeks gestation were used (Additional file 1: Table S3).

**Table 3:** **Associations between blood pressures (per 1-SD increase) and size at birth by maternal ethnicity** ^*^

**Maternal ethnicity**	**N**	**Peripheral systolic blood pressure (1 SD = 11.1 mmHg)**	**Peripheral diastolic blood pressure (1 SD = 8.3 mmHg)**	**Central systolic blood pressure (1 SD = 10.0 mmHg)**	**Central pulse pressure (1 SD = 6.5 mmHg)**
		**ß (95% CI)**	**ß (95% CI)**	**ß (95% CI)**	**ß (95% CI)**
**Weight (g)**					
Chinese	399	−49.10 (−89.63 to −8.56)	−37.74 (−76.82 to 1.33)	−52.12 (−91.19 to −13.11)	−26.49 (−62.65 to 9.67)
Indian	118	−17.37 (−98.58 to 63.84)	0.17 (−76.87 to 77.22)	−30.70 (−109.08 to 47.67)	−29.87 (−91.32 to 32.15)
Malay	196	−4.88 (−70.38 to 60.62)	−17.29 (−80.97 to 46.39)	−17.00 (−80.93 to 46.93)	−3.21 (−63.08 to 56.65)
*P* for interaction		0.96	0.78	0.98	0.96
**Length (cm)** ^**^					
Chinese	398	−0.11 (−0.35 to 0.12)	−0.14 (−0.37 to 0.08)	−0.16 (−0.39 to 0.06)	−0.05 (−0.26 to 0.16)
Indian	117	−0.14 (−0.54 to -.26)	−0.07 (−0.44 to 0.31)	−0.19 (−0.57 to 0.19)	−0.13 (−0.43 to 0.18)
Malay	196	−0.24 (−0.57 to 0.08)	0.05 (−0.27 to 0.37)	−0.24 (−0.56 to 0.08)	−0.38 (−0.67 to −0.08)
*P* for interaction		0.16	0.96	0.29	0.09
**Head circumference (cm)** ^**^					
Chinese	398	−0.13 (−0.28 to 0.01)	−0.13 (−0.27 to 0.00)	−0.17 (−0.31 to −0.04)	−0.08 (−0.21 to 0.05)
Indian	117	−0.04 (−0.32 to 0.23)	0.10 (−0.15 to 0.36)	−0.10 (−0.37 to 0.16)	−0.18 (−0.39 to 0.02)
Malay	196	0.03 (−0.19 to 0.26)	−0.09 (−0.31 to 0.13)	−0.00 (−0.22 to 0.22)	0.09 (−0.11 to 0.30)
*P* for interaction		0.69	0.81	0.63	0.18
**Placenta weight (g)** ^***^					
Chinese	392	−8.27 (−21.94 to 5.40)	−6.28 (−19.47 to 6.92)	−11.22 (−24.43 to 1.99)	−7.43 (−19.37 to 4.51)
Indian	115	−11.15 (−37.06 to 14.76)	−5.05 (−29.82 to 19.72)	−13.62 (−38.69 to 11.44)	−8.94 (−28.84 to 10.95)
Malay	192	−9.16 (−28.52 to 10.19)	−12.83 (−31.39 to 5.73)	−11.79 (−30.53 to 6.94)	−1.13 (−18.65 to 16.39)
*P* for interaction		0.93	0.80	0.89	0.97

SD, standard deviation; CI, confidence interval.

^*^Multiple linear regression models were used with adjustment for baby’s sex, gestation at delivery, maternal age, education, parity, smoking history, height, BMI at 27 weeks gestation, coffee consumption and depression.

^**^There were 2 women with missing information on length and head circumference.

^***^There were 14 women with missing information on placental weight.

**Table 4:** **Associations between blood pressures (per 1-SD increase) and size at birth by maternal BMI** ^*^

**Maternal BMI according to WHO classification**	**N**	**Peripheral systolic blood pressure (1 SD = 11.1 mmHg)**	**Peripheral diastolic blood pressure (1 SD = 8.3 mmHg)**	**Central systolic blood pressure (1 SD = 10.0 mmHg)**	**Central pulse pressure (1 SD = 6.5 mmHg)**
		**ß (95% CI)**	**ß (95% CI)**	**ß (95% CI)**	**ß (95% CI)**
**Weight (g)**					
BMI <25.0 kg/m^2^	329	−74.50 (−117.92 to −31.08)	−38.79 (−80.33 to 2.74)	−81.36 (−122.36 to −40.32)	−66.81 (−105.84 to −27.80)
BMI 25.0-29.9 kg/m^2^	259	−26.64 (−79.84 to 26.56)	−60.87 (−111.65 to −10.10)	−41.90 (−94.50 to 10.69)	13.56 (−31.22 to 58.35)
BMI ≥30.0 kg/m^2^	125	14.59 (−72.21 to 101.40)	80.71 (−8.57 to 170.01)	41.23 (−45.56 to 128.03)	−24.39 (−99.09 to 50.31)
*P* for interaction		0.06	0.02	0.02	0.02
**Length (cm)** ^**^					
BMI <25.0 kg/m^2^	327	−0.40 (−0.65 to −0.15)	−0.18 (−0.42 to 0.05)	−0.44 (−0.68 to −0.21)	−0.40 (−0.63 to −0.18)
BMI 25.0-29.9 kg/m^2^	259	0.04 (−0.25 to 0.33)	−0.29 (−0.56 to −0.01)	−0.09 (−0.38 to 0.19)	0.18 (−0.06 to 0.42)
BMI ≥30.0 kg/m^2^	125	−0.07 (−0.48 to 0.34)	0.51 (0.09 to 0.93)	0.12 (−0.29 to 0.54)	−0.31 (−0.66 to 0.04)
*P* for interaction		0.04	0.009	0.03	0.001
**Head circumference (cm)** ^**^					
BMI <25.0 kg/m^2^	327	−0.19 (−0.35 to −0.04)	−0.20 (−0.35 to −0.06)	−0.22 (−0.37 to −0.07)	−0.07 (−0.21 to 0.07)
BMI 25.0-29.9 kg/m^2^	259	−0.08 (−0.27 to 0.10)	−0.10 (−0.28 to 0.08)	−0.12 (−0.31 to 0.06)	−0.03 (−0.19 to 0.12)
BMI ≥30.0 kg/m^2^	125	0.17 (−0.11 to 0.45)	0.31 (0.02 to 0.59)	0.13 (−0.15 to 0.42)	−0.12 (−0.36 to 0.12)
*P* for interaction		0.04	0.004	0.05	0.87
**Placenta weight (g)** ^***^					
BMI <25.0 kg/m^2^	323	−17.72 (−32.74 to −2.69)	−11.45 (−25.67 to 2.76)	−20.54 (−34.79 to −6.29)	−14.05 (−27.27 to −0.83)
BMI 25.0-29.9 kg/m^2^	256	−5.85 (−21.75 to 10.06)	−9.62 (−24.95 to 5.71)	−10.14 (−25.91 to 5.62)	−1.76 (−15.11 to 11.58)
BMI ≥30.0 kg/m^2^	120	−0.26 (−27.15 to 26.63)	4.16 (−23.54 to 31.87)	1.23 (−25.54 to 27.99)	−2.27 (−25.05 to 20.51)
*P* for interaction		0.18	0.49	0.15	0.25

SD, standard deviation; CI, confidence interval; BMI, body mass index.

^*^Multiple linear regression models were used with adjustment for baby’s sex, gestation at delivery, maternal age, ethnicity, education, parity, smoking history, height, BMI at 27 weeks gestation, coffee consumption and depression.

^**^There were 2 women with missing information on length and head circumference.

^***^There were 14 women with missing information on placental weight.

To further account for the influence from gestational age, we used gestational age adjusted SD scores of size at birth as the outcomes, and similar findings were observed (Additional file 1: Tables S4-S6). Using binary variables of birth weight, we found that higher peripheral and central blood pressures were associated with higher odds for low birth weight and small for gestational age infants (Table 5). Tests for interactions between maternal ethnicity and blood pressures were not significant (data not shown), whereas the interactions between maternal BMI category and blood pressures (peripheral SBP, central SBP and PP) were borderline significant for small for gestational age (p *=* 0.04 to 0.06), and the interaction between maternal BMI category and central PP was significant for low birth weight (p = 0.01). Again, the odds ratios were generally stronger in normal weight women compared to overweight/obese women (Table 5).

**Table 5:** **The association between maternal blood pressures (per 1-SD increase) and low birth weight and small for gestational age**

	**N**	**Peripheral systolic blood pressure (1 SD = 11.1 mmHg)**	**Peripheral diastolic blood pressure (1 SD = 8.3 mmHg)**	**Central systolic blood pressure (1 SD = 10.0 mmHg)**	**Central pulse pressure (1 SD = 6.5 mmHg)**
		**OR (95% CI)**	**OR (95% CI)**	**OR (95% CI)**	**OR (95% CI)**
**Low birth weight**					
All women^*^	713	1.64 (1.12 to 2.41)	1.82 (1.27 to 2.61)	1.85 (1.29 to 2.67)	1.17 (0.85 to 1.62)
BMI categories^**^					
BMI <25.0 kg/m^2^	329	2.12 (1.27 to 3.53)	1.70 (1.06 to 2.72)	2.41 (1.45 to 3.99)	1.82 (1.15 to 2.88)
BMI ≥25.0 kg/m^2^	384	1.09 (0.60 to 1.99)	1.88 (1.09 to 3.24)	1.28 (0.73 to 2.24)	0.62 (0.34 to 1.13)
*P* for interaction		0.11	0.86	0.14	0.01
**Small for gestational age**					
All women^*^	713	1.58 (1.16 to 2.14)	1.41 (1.06 to 1.89)	1.70 (1.27 to 2.28)	1.36 (1.05 to 1.76)
BMI categories^**^					
BMI <25.0 kg/m^2^	329	1.88 (1.24 to 2.86)	1.43 (0.97 to 2.09)	2.01 (1.35 to 3.00)	1.74 (1.19 to 2.53)
BMI ≥25.0 kg/m^2^	384	1.17 (0.72 to 1.92)	1.27 (0.79 to 2.03)	1.26 (0.79 to 2.03)	1.04 (0.69 to 1.55)
*P* for interaction		0.04	0.44	0.06	0.04

SD, standard deviation; OR, odds ratio; CI, confidence interval.

^*^Multiple logistic regression models were used with adjustment for baby’s sex, gestation at delivery, maternal age, ethnicity, education, parity, smoking history, height, BMI at 27 weeks gestation, coffee consumption and depression. In the analysis of small for gestational age, gestation weeks at delivery was not adjusted for.

^**^Adjusted baby’s sex, gestation at delivery, maternal age, ethnicity, education, parity, smoking history, height, BMI at 27 weeks gestation, coffee consumption and depression. In the analysis for small for gestational age, gestation at delivery was not adjusted for. Women with BMI 25.0-29.9 or ≥ 30.0 kg/m^2^ were grouped together due to problems with small sample size and model convergence.

## Discussion

We found associations between higher maternal blood pressures and smaller offspring. Maternal adiposity modified the associations with stronger inverse associations in normal weight women than their overweight/obese counterparts. No significant effect modification by ethnicity were found, although Chinese women with higher blood pressures tended to have smaller offspring.

Our finding of an inverse association between maternal blood pressures and offspring size at birth is consistent with previous studies [3,5-9,13-17]. For example, Bakker et al. [5] reported that per one-SD increase in SBP and DBP at mean gestation of 30.2 weeks (range 28.4 to 32.9 weeks) was associated with 16.9 g and 50.6 g lower birth weight, respectively. Among non-hypertensive women, higher peripheral blood pressures (range of gestation 26 to 39 weeks) were also associated with lower birth weight [9]; and higher central blood pressures (range of gestation 22 to 39 weeks) were associated with lower birth weight [13,14].

However, there are studies with conflicting results. For example, two previous studies measuring DBP from 34 weeks gestation onwards [12] and the average of SBP and DBP during pregnancy [10] have described a u-shaped association with birth weight. In another perinatal cohort study, DBP measured between 15 to 24 weeks gestation were not found to be significantly associated with birth weight [11]. These studies [10,12] were based on retrospective cohorts design that utilized blood pressure information collected under clinical context whereas studies that reported inverse associations were prospective cohort design with blood pressure information collected by the study investigators. Furthermore, the DBP measures reported in these retrospective cohort studies [11,12] were based on either Korotkoff Phase IV or V from standard mercury sphygmomanometer compared to the automated oscillometric device used in other studies [5,6] or Spacelabs blood pressure monitor [7,8]. Varying DBP measures arising from the different Korotkoff phases [24] and blood pressure devices could have contributed to the conflicting results.

Some studies have suggested that central blood pressures may be more relevant to size at birth than the conventional peripheral blood pressures, because blood pressure differences were more pronounced in central than peripheral measures [13,14]. However, in the current analysis, we found similar effect estimates between central and peripheral blood pressures, which is consistent with an earlier report by Elvan-Taspinar et al. [9]. Although central blood pressures may be better markers for arterial stiffness [23,25], the role of central and peripheral blood pressures in relation to offspring birth size have yet to be ascertained due to the limited and divergent literature.

The exact mechanisms linking higher maternal blood pressures and smaller offspring are unclear. Several studies have observed that women with preeclampsia and low birth weight offsprings share a common link in placental dysfunction [26-29]. But whether placental dysfunction precedes maternal hypertension, or that it arises from maternal hypertension as a consequence of pre-existing maternal predisposition to endothelial dysfunction, current literature is still controversial [6,26,27,29]. Although the exact mechanism is unclear, higher maternal blood pressure could be a feature shared by both endothelial dysfunction and placental dysfunction, as both entities are not mutually exclusive [28].

A previous study by Lydakis et al. [15] found that the relationship between higher maternal blood pressures and lower birth weight was stronger in Asian Indians than white or black women, but no studies have yet tested the ethnic differences within Asian women. Our study is the first in its kind in three Asian ethnicities, and we observed no significant ethnic differences in the association between blood pressure and birth outcomes. Our results of inverse associations were also supported by some studies in Asian women, where pre-eclampsia was associated with increased risk to small for gestational age in Chinese women [16] and lower birth weight in Indian women [17]. However, we cannot exclude the possibility of ethnic differences due to the smaller subgroups of Indian and Malay women in our cohort and therefore was not powered to detect effect modification by maternal ethnicity. Our exploratory analysis on the ethnic differences in the relations between blood pressures and birth size were among the first few in literature and future studies are still needed to further explore the potential ethnic differences.

Our finding on the effect modification by maternal obesity is consistent with literature that lean or normal weight women with higher blood pressures have smaller offspring compared to their obese counterparts [6]. The effect modification by maternal obesity on fetal growth restriction, may be due to the higher fetal nutrient supply in obese women [30], and the overall effect of maternal obesity and blood pressures on birth weight may be dependent on the balance of these factors [6,29].

There are several strengths of our study. The prospective design enabled the evaluation of a comprehensive information on offspring size at birth and a wide range of potential confounding factors. Peripheral blood pressure and radial pulse wave were measured in a detailed and standardized approach, thereby minimizing inter-rater measurement errors. Various sensitivity analyses suggested that our results were robust.

We are aware of several limitations. First, we excluded 38.6% of the GUSTO participants due to missing information on the exposures and covariates. However, we deemed that the selection bias was unlikely to change our results based on the sensitivity analysis using imputed data (Additional file 1: Table S2). Second, we did not have data on first and third trimester blood pressure, and thus were unable to assess trimester specific blood pressure changes during pregnancy in relation to size at birth. Thirdly, the use of maternal BMI at 27 weeks gestation may be affected by misclassication due to the growing fetus and fluid accumulation. We chose to use mid-pregnancy BMI instead of pre-pregnancy BMI because the latter measure was self-reported and thus susceptible to information bias, and about 5% of the women did not report their pre-pregnancy weights. Due to the lack of pregnancy-specific classification for obesity, we have used the WHO cut-offs for non-pregnant adults in our study. However, our sensitivity analysis of using tertiles of BMI suggested that the interaction with BMI categories was robust. We did not measure maternal weight before delivery, and could not know whether the relation between blood pressure and birth size outcomes would be changed if total weight gain during pregnancy was adjusted in the model. Our results may also be affected by residual confounding from coffee intake as it was self-reported, and unmeasured confounding factors, like diet and physical activity, are possible to explain our results.

## Conclusion

In conclusion, our results provide further evidence that higher second trimester blood pressures are associated with smaller offspring, with a stronger association among normal weight women. Therefore, routine antenatal monitoring of maternal blood pressures are clinically relevant and important practice, and may have a positive impact on offspring size at birth, particularly in normal weight women.

## Additional file

Additional file 1: Table S1 Maternal characteristics by study inclusion to the present study. **Table S2.** Sensitivity analysis for per 1-SD increase in maternal blood pressures and size at birth. A series of analysis using imputed data for missing information on maternal blood pressure; varying the adjustment for maternal confounders such as gestational diabetes, maternal adiposity using pre-pregnancy BMI and rate of weight gain per week; and in a subgroup of women with peripheral systolic blood pressures less than 140 mmHg and diastolic blood pressures less than 90 mmHg. **Table S3.** Per 1-SD increase in maternal blood pressures and size at birth by maternal BMI in tertiles. **Table S4.** Per 1-SD increase in maternal blood pressures and gestational age adjusted SD scores of birth size outcomes. **Table S5.** Per 1-SD increase in maternal blood pressures and gestational age adjusted SD scores of birth size outcomes by maternal ethnicity. **Table S6.** Per 1-SD increase in maternal blood pressures and gestational age adjusted SD scores of birth size outcomes by maternal BMI according to WHO classification.

## Abbreviations

SBP: Systolic blood pressure
DBP: Diastolic blood pressure
PP: Pulse pressure
GUSTO: **G**rowing **U**p in **S**ingapore **T**owards Healthy **O**utcomes
BMI: Body mass index
SD: Standard deviation
CI: Confidence interval

## References

[1] Kramer MS (1987). Determinants of low birth weight: methodological assessment and meta-analysis. Bull World Health Organ.

[2] Mayer C, Joseph KS (2013). Fetal growth: a review of terms, concepts and issues relevant to obstetrics. Ultrasound Obstet Gynecol.

[3] Odegard RA, Vatten LJ, Nilsen ST, Salvesen KA, Austgulen R (2000). Preeclampsia and fetal growth. Obstet Gynecol.

[4] Rahman LA, Hairi NN, Salleh N (2008). Association between pregnancy induced hypertension and low birth weight; a population based case–control study. Asia Pac J Public Health.

[5] Bakker R, Steegers EA, Hofman A, Jaddoe VW (2011). Blood pressure in different gestational trimesters, fetal growth, and the risk of adverse birth outcomes: the generation R study. Am J Epidemiol.

[6] Romundstad PR, Davey Smith G, Nilsen TI, Vatten LJ (2007). Associations of prepregnancy cardiovascular risk factors with the offspring’s birth weight. Am J Epidemiol.

[7] Churchill D, Perry IJ, Beevers DG (1997). Ambulatory blood pressure in pregnancy and fetal growth. Lancet.

[8] Waugh J, Perry IJ, Halligan AW, De Swiet M, Lambert PC, Penny JA, Taylor DJ, Jones DR, Shennan A (2000). Birth weight and 24-hour ambulatory blood pressure in nonproteinuric hypertensive pregnancy. Am J Obstet Gynecol.

[9] Elvan-Taspinar A, Franx A, Bots ML, Koomans HA, Bruinse HW (2005). Arterial stiffness and fetal growth in normotensive pregnancy. Am J Hypertens.

[10] Yadav H, Lee N (2013). Maternal factors in predicting low birth weight babies. Med J Malaysia.

[11] Zhang J, Klebanoff MA (2001). Low blood pressure during pregnancy and poor perinatal outcomes: an obstetric paradox. Am J Epidemiol.

[12] Steer PJ, Little MP, Kold-Jensen T, Chapple J, Elliott P: **Maternal blood pressure in pregnancy, birth weight, and perinatal mortality in first births: prospective study.** *BMJ* 2004, **329**(7478):1312. 10.1136/bmj.38258.566262.7C PMC534837 15561733

[13] Tomimatsu T, Fujime M, Kanayama T, Mimura K, Koyama S, Kanagawa T, Kimura T (2013). Maternal arterial stiffness in normotensive pregnant women who subsequently deliver babies that are small for gestational age. Eur J Obstet Gynecol Reprod Biol.

[14] Khan F, Mires G, Macleod M, Belch JJ (2010). Relationship between maternal arterial wave reflection, microvascular function and fetal growth in normal pregnancy. Microcirculation.

[15] Lydakis C, Beevers DG, Beevers M, Lip GY (1998). Obstetric and neonatal outcome following chronic hypertension in pregnancy among different ethnic groups. QJM.

[16] Xiong X, Fraser WD (2004). Impact of pregnancy-induced hypertension on birthweight by gestational age. Paediatr Perinat Epidemiol.

[17] Dhall K, Bagga R (1995). Maternal determinants of birth weight of north Indian babies. Indian J Pediatr.

[18] Katz J, Lee AC, Kozuki N, Lawn JE, Cousens S, Blencowe H, Ezzati M, Bhutta ZA, Marchant T, Willey BA, Adair L, Barros F, Baqui AH, Christian P, Fawzi W, Gonzalez R, Humphrey J, Huybregts L, Kolsteren P, Mongkolchati A, Mullany LC, Ndyomugyenyi R, Nien JK, Osrin D, Roberfroid D, Sania A, Schmiegelow C, Silveira MF, Tielsch J, Vaidya A (2013). Mortality risk in preterm and small-for-gestational-age infants in low-income and middle-income countries: a pooled country analysis. Lancet.

[19] Soh SE, Tint MT, Gluckman PD, Godfrey KM, Rifkin-Graboi A, Chan YH, Stunkel W, Holbrook JD, Kwek K, Chong YS, Saw SM, GUSTO Study Group (2014). Cohort profile: Growing Up in Singapore Towards healthy Outcomes (GUSTO) birth cohort study. Int J Epidemiol.

[20] Williams B, Lacy PS, Yan P, Hwee C-N, Liang C, Ting C-M (2011). Development and validation of a novel method to derive central aortic systolic pressure from the radial pressure waveform using an N-point moving average method. J Am Coll Cardiol.

[21] Alberti KG, Zimmet PZ (1998). Definition, diagnosis and clssification of diabetes mellitus and its comlications. Part 1: diagnosis and classification of diabetes mellitus provisional report of a WHO consultation. Diabetes Med.

[22] WHO (2000). Obesity: preventing and managing the global epidemic. Report of a WHO Consultation. WHO Technical Report Series 894.

[23] Williams B, Lacy PS (2010). Central haemodynamics and clinical outcomes: going beyond brachial blood pressure?. Eur Heart J.

[24] Shennan A, Gupta M, Halligan A, Taylor DJ, de Swiet M (1996). Lack of reproducibility in pregnancy of Korotkoff phase IV as measured by mercury sphygmomanometry. Lancet.

[25] Fujime M, Tomimatsu T, Okaue Y, Koyama S, Kanagawa T, Taniguchi T, Kimura T (2012). Central aortic blood pressure and augmentation index during normal pregnancy. Hypertens Res.

[26] Kaufmann P, Black S, Huppertz B (2003). Endovascular trophoblast invasion: implications for the pathogenesis of intrauterine growth retardation and preeclampsia. Biol Reprod.

[27] Hafner E, Metzenbauer M, Hofinger D, Munkel M, Gassner R, Schuchter K, Dillinger-Paller B, Philipp K (2003). Placental growth from the first to the second trimester of pregnancy in SGA-foetuses and pre-eclamptic pregnancies compared to normal foetuses. Placenta.

[28] Everett TR, Lees CC (2012). Beyond the placental bed: placental and systemic determinants of the uterine artery Doppler waveform. Placenta.

[29] Ness RB, Sibai BM (2006). Shared and disparate components of the pathophysiologies of fetal growth restriction and preeclampsia. Am J Obstet Gynecol.

[30] Harmon KA, Gerard L, Jensen DR, Kealey EH, Hernandez TL, Reece MS, Barbour LA, Bessesen DH (2011). Continuous glucose profiles in obese and normal-weight pregnant women on a controlled diet: metabolic determinants of fetal growth. Diabetes Care.

