# Supplementary material for: The association between maternal blood pressures and offspring size at birth in Southeast Asian women
Source: BMC Pregnancy Childbirth. 2014 Dec 2;14:403. doi: 10.1186/s12884-014-0403-1 (PMC4259008; doi:10.1186/s12884-014-0403-1)
Supplement: Additional file 1: Table S1 — Maternal characteristics by study inclusion to the present study. Table S2. Sensitivity analysis for per 1-SD increase in maternal blood pressures and size at birth. A series of analysis using imputed data for missing information on maternal blood pressure; varying the adjustment for maternal confounders such as gestational diabetes, maternal adiposity using pre-pregnancy BMI and rate of weight gain per week; and in a subgroup of women with peripheral systolic blood pressures less than 140 mmHg and diastolic blood pressures less than 90 mmHg. Table S3. Per 1-SD increase in maternal blood pressures and size at birth by maternal BMI in tertiles. Table S4. Per 1-SD increase in maternal blood pressures and gestational age adjusted SD scores of birth size outcomes. Table S5. Per 1-SD increase in maternal blood pressures and gestational age adjusted SD scores of birth size outcomes by maternal ethnicity. Table S6. Per 1-SD increase in maternal blood pressures and gestational age adjusted SD scores of birth size outcomes by maternal BMI according to WHO classification. [file 12884_2014_403_MOESM1_ESM.doc]

Additional file 1: **Table S1. Maternal Characteristics by Study Inclusion to the Present Study**

| **Maternal Characteristics** | **Included in study** | | **Excluded from study** | | ***P**** |
| --- | --- | --- | --- | --- | --- |
|  | **n** | Mean (SD) or % | **n** | Mean (SD) or % |  |
| Peripheral systolic blood pressure (mmHg) | 713 | 109.3 (11.1) | 116 | 110.6 (11.7) | 0.25 |
| Peripheral diastolic blood pressure (mmHg) | 713 | 66.7 (8.2) | 116 | 67.4 (9.9) | 0.38 |
| Central systolic blood pressure (mmHg) | 713 | 96.7 (9.9) | 116 | 97.9 (10.9) | 0.20 |
| Central pulse pressure (mmHg) | 713 | 30.0 (6.5) | 116 | 30.6 (6.8) | 0.41 |
| Age at booking (years) |  |  |  |  | 0.18 |
| 1^st^ quartile (18-26) | 162 | 22.7% | 119 | 27.7% |  |
| 2^nd^ quartile (27-29) | 148 | 20.8% | 83 | 19.4% |  |
| 3^rd^ quartile (30-33) | 204 | 28.6% | 104 | 24.2% |  |
| 4^th^ quartile (34-46) | 199 | 27.9% | 123 | 28.7% |  |
| Race |  |  |  |  | 0.23 |
| Chinese | 399 | 55.9% | 222 | 51.8% |  |
| Indian | 118 | 16.6% | 87 | 20.3% |  |
| Malay | 196 | 27.5% | 120 | 27.9% |  |
| Education |  |  |  |  | 0.37 |
| Primary-Secondary | 229 | 32.1% | 138 | 32.8% |  |
| Post-Secondary | 245 | 34.4% | 158 | 37.5% |  |
| Tertiary | 239 | 33.5% | 125 | 29.7% |  |
| Smoking Status |  |  |  |  | 0.47 |
| Never smoker | 618 | 86.7% | 290 | 85.0% |  |
| Ever smoker | 95 | 13.3% | 51 | 15.0% |  |
| Coffee Consumption |  |  |  |  | 0.42 |
| No | 369 | 51.8% | 186 | 54.4% |  |
| Yes | 344 | 48.2% | 156 | 45.6% |  |
| Parity |  |  |  |  | 0.84 |
| Nulliparous | 311 | 43.6% | 147 | 41.8% |  |
| Primiparous | 246 | 34.5% | 124 | 35.2% |  |
| Multiparous | 156 | 21.9% | 81 | 23.0% |  |
| Gestational Diabetes |  |  |  |  | 0.27 |
| No | 540 | 81.4% | 304 | 84.2% |  |
| Yes | 123 | 18.6% | 57 | 15.8% |  |
| Pre-pregnancy BMI (kg/m^2^) |  |  |  |  | 0.58 |
| <25.0 | 518 | 76.4% | 243 | 73.4% |  |
| 25.0-29.9 | 113 | 16.7% | 62 | 18.7% |  |
| ≥30.0 | 47 | 6.9% | 26 | 7.8% |  |
| 2^nd^ trimester BMI (kg/m^2^) |  |  |  |  | 0.19 |
| <25.0 | 329 | 46.1% | 180 | 49.2% |  |
| 25.0-29.9 | 259 | 36.3% | 113 | 30.9% |  |
| ≥30.0 | 125 | 17.5% | 73 | 19.9% |  |
| Rate of weight gain at 27 weeks (kg/week) |  |  |  |  | 0.46 |
| 1^st^ tertile (-0.42 – 0.25) | 227 | 33.5% | 107 | 33.0% |  |
| 2^nd^ tertile (0.26 – 0.37) | 218 | 32.2% | 116 | 35.8% |  |
| 3^rd^ tertile (0.38 – 1.38) | 233 | 34.4% | 101 | 31.2% |  |
| Depression |  |  |  |  | 0.16 |
| Not depressed | 631 | 88.5% | 300 | 85.5% |  |
| Depressed | 82 | 11.5% | 51 | 14.5% |  |
| Gestation at delivery (weeks) | 713 | 38.8 (1.4) | 352 | 38.6 (1.8) | 0.006 |
| Size at birth |  |  |  |  |  |
| Weight (g) | 713 | 3113.5 (435.0) | 350 | 3034.2 (478.2) | 0.007 |
| Length (cm) | 711 | 48.7 (2.2) | 351 | 48.2 (2.4) | 0.001 |
| Head circumference (cm) | 711 | 33.4 (1.36) | 350 | 33.2 (1.6) | 0.006 |
| Placenta weight (g) | 699 | 585.3 (118.9) | 346 | 561.5 (124.0) | 0.003 |

BMI, body mass index.

Data are mean (standard deviation) or % as specified.

*Using Student *t* test for continuous variables or χ^2^ test for categorical variables.

**Table S2. Sensitivity Analysis for per 1-SD Increase in Maternal Blood Pressures and Size at Birth**

|  | **N** | **Peripheral Systolic**  **Blood Pressure**  **(1 SD = 11.1 mmHg)** | **Peripheral Diastolic**  **Blood Pressure**  **(1 SD = 8.3 mmHg)** | **Central Systolic**  **Blood Pressure**  **(1 SD = 10.0 mmHg)** | **Central Pulse Pressure**  **(1 SD = 6.5 mmHg)** |
| --- | --- | --- | --- | --- | --- |
|  |  | **ß (95% CI)** | **ß (95% CI)** | **ß (95% CI)** | **ß (95% CI)** |
| **Weight (g)** |  |  |  |  |  |
| Main Model | 713 | -35.56 (-66.57 to -4.54) | -25.13 (-55.36 to 5.09) | -40.52 (-70.66 to -10.37) | -24.10 (-51.24 to 3.03) |
| Main Model + GDM* | 663 | -34.47 (-66.93 to -1.99) | -32.77 (-64.51 to -1.03) | -41.72 (-73.05 to -10.38) | -18.99 (-47.71 to 9.71) |
| Main Model among non-hypertensive women | 705 | -36.25 (-68.02 to -4.47) | -30.16 (-61.28 to 0.97) | -42.63 (-73.65 to -11.61) | -20.83 (-48.31 to 6.64) |
| Main Model using multiple imputation | 922 | -34.00 (-65.25 to -2.75) | -24.37 (-53.15 to 4.41) | -38.43 (-67.47 to -9.39) | -21.09 (-48.67 to 6.49) |
| Main Model with pre-pregnancy BMI & rate of weight gain | 678 | -35.05 (-66.66 to -3.43) | -21.19 (-51.69 to 9.33) | -40.14 (-70.79 to -9.49) | -27.21 (-54.67 to 0.25) |
| **Length (cm)**** |  |  |  |  |  |
| Main Model | 711 | -0.16 (-0.32 to 0.01) | -0.10 (-0.27 to 0.06) | -0.19 (-0.36 to -0.03) | -0.14 (-0.28 to 0.01) |
| Main Model + GDM* | 661 | -0.17 (-0.35 to 0.00) | -0.14 (-0.31 to 0.04) | -0.21 (-0.38 to -0.04) | -0.13 (-0.28 to 0.03) |
| Main Model among non-hypertensive women | 703 | -0.18 (-0.36 to -0.01) | -0.14 (-0.31 to 0.02) | -0.23 (-0.40 to -0.06) | -0.13 (-0.28 to 0.02) |
| Main Model using multiple imputation | 921 | -0.16 (-0.32 to 0.01) | -0.11 (-0.27 to 0.05) | -0.21 (-0.37 to -0.06) | -0.12 (-0.26 to 0.02) |
| Main Model with pre-pregnancy BMI & rate of weight gain | 676 | -0.15 (-0.32 to 0.02) | -0.10 (-0.27 to 0.06) | -0.19 (-0.36 to -0.02) | -0.12 (-0.28 to 0.02) |
| **Head circumference (cm)**** | |  |  |  |  |
| Main Model | 711 | -0.09 (-0.19 to 0.02) | -0.08 (-0.18 to 0.02) | -0.12 (-0.23 to -0.02) | -0.07 (-0.16 to 0.02) |
| Main Model + GDM* | 661 | -0.07 (-0.19 to 0.04) | -0.07 (-0.18 to 0.04) | -0.10 (-0.21 to 0.01) | -0.05 (-0.15 to 0.05) |
| Main Model among non-hypertensive women | 703 | -0.10 (-0.21 to 0.01) | -0.10 (-0.21 to 0.01) | -0.14 (-0.25 to -0.04) | -0.07 (-0.17 to 0.02) |
| Main Model using multiple imputation | 921 | -0.08 (-0.20 to 0.03) | -0.08 (-0.18 to 0.03) | -0.12 (-0.22 to -0.01) | -0.06 (-0.15 to 0.04) |
| Main Model with pre-pregnancy BMI & rate of weight gain | 676 | -0.09 (-0.20 to 0.02) | -0.08 (-0.18 to 0.03) | -0.12 (-0.23 to -0.02) | -0.07 (-0.17 to 0.02) |
| **Placenta weight (g)***** |  |  |  |  |  |
| Main Model | 699 | -8.78 (-18.74 to 1.19) | -6.94 (-16.63 to 2.76) | -11.16 (-20.85 to -1.47) | -6.44 (-15.04 to 2.16) |
| Main Model + GDM* | 650 | -11.48 (-22.05 to -0.92) | -9.19 (-19.51 to 1.13) | -13.27 (-23.46 to -3.07) | -7.12 (-16.32 to 2.08) |
| Main Model among non-hypertensive women | 691 | -8.93 (-19.17 to 1.31) | -7.70 (-17.72 to 2.32) | -11.54 (-21.54 to -1.54) | -5.92 (-14.65 to 2.80) |
| Main Model using multiple imputation | 908 | -8.04 (-18.21 to 2.13) | -8.23 (-17.39 to 0.93) | -11.23 (-21.31 to 1.14) | -5.64 (-13.92 to 2.65) |
| Main Model with pre-pregnancy BMI & rate of weight gain | 666 | -11.15 (-21.45 to -0.85) | -7.79 (17.70 to 2.12) | -13.01 (-22.99 to -3.02) | -7.59 (-16.43 to 1.24) |

SD, standard deviation; CI, confidence interval; GDM, gestational diabetes; IOM, Institute of Medicine

* There were 50 women with missing information on gestational diabetes

** There were 2 women with missing information on length and head circumference.

*** There were 14 women with missing information on placental weight.

Main model was derived from multiple linear regression with adjustment for baby's sex, gestation at delivery, maternal age, ethnicity, education, parity, smoking history, maternal BMI at 27 weeks gestation, height, coffee consumption and depression.

Main Model + GDM: adjusted for covariates in Main Model with additional adjustment for gestational diabetes.

Main Model among non-hypertensive women: the analysis was done in women with peripheral systolic blood pressures less than 140 mmHg and diastolic blood pressures less than 90 mmHg.

Main Model using multiple imputation: missing data in blood pressures were imputed from model with maternal age, ethnicity, education, parity, smoking history, BMI at 27 weeks gestation, height, gestational diabetes and the respective outcome variable.

Main Model with pre-pregnancy BMI is adjusted for the same covariates in Main Model except for maternal BMI at 27 weeks gestation which was replaced by pre-pregnancy BMI and rate of weight gain per week.

**Table S3. Per 1-SD Increase in Maternal Blood Pressures and Size at Birth by Maternal BMI in Tertiles***

| **Maternal BMI in Tertiles** | **N** | **Peripheral Systolic Blood Pressure**  **(1 SD = 11.1 mmHg)** | **Peripheral Diastolic Blood Pressure**  **(1 SD = 8.3 mmHg)** | **Central Systolic Blood Pressure**  **(1 SD = 10.0 mmHg)** | **Central Pulse Pressure**  **(1 SD = 6.5 mmHg)** |
| --- | --- | --- | --- | --- | --- |
|  |  | **ß (95% CI)** | **ß (95% CI)** | **ß (95% CI)** | **ß (95% CI)** |
| **Weight (g)** |  |  |  |  |  |
| 1^st^ Tertile | 234 | -89.57 (-142.41 to -36.73) | -32.09 (-82.41 to 18.23) | -95.6 (-145.66 to -45.54) | -84.53 (-129.73 to -39.33) |
| 2^nd^ Tertile | 244 | -25.05 (-77.64 to 27.53) | -53.05 (-103.05 to -3.05) | -26.05 (-76.72 to 24.62) | 24.38 (-21.83 to 70.59) |
| 3^rd^ Tertile | 235 | 2.21 (-57.05 to 61.47) | 14.56 (-44.92 to 74.04) | -6.28 (-65.48 to 52.92) | -21.13 (-72.20 to 29.93) |
| *P* for interaction |  | 0.03 | 0.09 | 0.03 | 0.008 |
| **Length (cm)**** |  |  |  |  |  |
| 1^st^ Tertile | 233 | -0.54 (-0.85 to -0.22) | -0.14 (-0.45 to 0.16) | -0.58 (-0.88 to -0.28) | -0.57 (-0.84 to -0.30) |
| 2^nd^ Tertile | 243 | 0.08 (-0.21 to 0.37) | -0.27 (-0.54 to 0.00) | -0.01 (-0.29 to 0.26) | 0.28 (0.03 to 0.53) |
| 3^rd^ Tertile | 235 | -0.03 (-0.33 to 0.27) | 0.18 (-0.12 to 0.48) | 0.00 (-0.30 to 0.30) | -0.17 (-0.43 to 0.09) |
| *P* for interaction |  | 0.02 | 0.06 | 0.006 | 0.0001 |
| **Head circumference (cm)**** | | |  |  |  |
| 1^st^ Tertile | 233 | -0.31 (-0.49 to -0.12) | -0.22 (-0.40 to -0.05) | -0.30 (-0.48 to -0.12) | -0.13 (-0.29 to 0.03) |
| 2^nd^ Tertile | 243 | -0.06 (-0.24 to 0.13) | -0.12 (-0.30 to 0.06) | -0.10 (-0.28 to 0.08) | 0.01 (-0.16 to 0.17) |
| 3^rd^ Tertile | 235 | 0.08 (-0.12 to 0.28) | 0.11 (-0.09 to 0.31) | 0.01 (-0.19 to 0.21) | -0.10 (-0.27 to 0.08) |
| *P* for interaction |  | 0.005 | 0.009 | 0.02 | 0.64 |
| **Placenta weight (g)***** | |  |  |  |  |
| 1^st^ Tertile | 230 | -28.89 (-47.19 to -10.59) | -16.57 (-33.81 to 0.66) | -29.41 (-46.89 to -11.93) | -17.04 (-32.46 to -1.63) |
| 2^nd^ Tertile | 240 | 6.60 (-9.09 to 22.29) | -6.38 (-21.45 to 8.68) | 0.12 (-15.04 to 15.28) | 7.06 (-6.71 to 20.82) |
| 3^rd^ Tertile | 229 | -8.07 (-26.95 to 10.81) | -1.07 (-19.94 to 17.81) | -9.83 (-28.60 to 8.95) | -10.18 (-26.30 to 5.94) |
| *P* for interaction |  | 0.02 | 0.22 | 0.04 | 0.08 |

SD, standard deviation; CI, confidence interval; BMI, body mass index.

* Multiple linear regression models were used with adjustment for baby's sex, gestation at delivery, maternal age, ethnicity, education, parity, smoking history, height, BMI at 27 weeks gestation, coffee consumption and depression.

** There were 2 women with missing information on length and head circumference.

*** There were 14 women with missing information on placental weight.

**Table S4. Per 1-SD Increase in Maternal Blood Pressures and Gestational Age Adjusted SD Scores of Birth Size Outcomes***

| **Measures of Size at Birth** | **N** | **Peripheral Systolic Blood Pressure**  **(1 SD = 11.1 mmHg)** | **Peripheral Diastolic Blood Pressure**  **(1 SD = 8.3 mmHg)** | **Central Systolic Blood Pressure**  **(1 SD = 10.0 mmHg)** | **Central Pulse Pressure**  **(1 SD = 6.5 mmHg)** |
| --- | --- | --- | --- | --- | --- |
|  |  | **ß (95% CI)** | **ß (95% CI)** | **ß (95% CI)** | **ß (95% CI)** |
| Weight (SD) | 713 | -0.08 (-0.17 to 0.00) | -0.06 (-0.14 to 0.02) | -0.10 (-0.18 to -0.02) | -0.06 (-0.13 to 0.01) |
| Length (SD)** | 711 | -0.07 (-0.15 to 0.02) | -0.05 (-0.13 to 0.04) | -0.08 (-0.17 to -0.003) | -0.06 (-0.14 to 0.02) |
| Head circumference (SD)** | 711 | -0.06 (-0.14 to 0.02) | -0.05 (-0.13 to 0.03) | -0.09 (-0.17 to -0.01) | -0.06 (-0.13 to 0.01) |
| Placental weight (SD)*** | 699 | -0.07 (-0.16 to 0.01) | -0.06 (-0.14 to 0.02) | -0.09 (-0.18 to -0.01) | -0.05 (-0.12 to 0.02) |

SD, standard deviation; CI, confidence interval.

* Measures of size at birth were estimated as gestational age adjusted standard deviation scores. Multiple linear regression models were used with adjustment for baby's sex, maternal age, ethnicity, education, parity, smoking history, height, BMI at 27 weeks gestation, coffee consumption and depression.

** There were 2 women with missing information on length and head circumference.

*** There were 14 women with missing information on placental weight.

**Table S5. Per 1-SD Increase in Maternal Blood Pressures and Gestational Age Adjusted SD Scores of Birth Size Outcomes** by Maternal Ethnicity*

| **Maternal Ethnicity** | **N** | **Peripheral Systolic Blood Pressure**  **(1 SD = 11.1 mmHg)** | **Peripheral Diastolic Blood Pressure**  **(1 SD = 8.3 mmHg)** | **Central Systolic Blood Pressure**  **(1 SD = 10.0 mmHg)** | **Central Pulse Pressure**  **(1 SD = 6.5 mmHg)** |
| --- | --- | --- | --- | --- | --- |
|  |  | **ß (95% CI)** | **ß (95% CI)** | **ß (95% CI)** | **ß (95% CI)** |
| **Weight (SD)** |  |  |  |  |  |
| Chinese | 399 | -0.12 (-0.23 to -0.01) | -0.10 (-0.21 to 0.001) | -0.14 (-0.24 to -0.03) | -0.07 (-0.17 to 0.03) |
| Indian | 118 | 0.01 (-0.20 to 0.22) | 0.02 (-0.18 to 0.22) | -0.05 (-0.26 to 0.16) | -0.06 (-0.23 to 0.09) |
| Malay | 196 | -0.02 (-0.19 to 0.15) | -0.04 (-0.21 to 0.13) | -0.05 (-0.22 to 0.12) | -0.02 (-0.18 to 0.14) |
| *P* for interaction |  | 0.90 | 0.80 | 0.96 | 0.98 |
| **Length (SD)**** |  |  |  |  |  |
| Chinese | 398 | -0.04 (-0.16 to 0.07) | -0.07 (-0.19 to 0.04) | -0.07 (-0.19 to 0.04) | -0.02 (-0.12 to 0.09) |
| Indian | 117 | -0.03 (-0.23 to 0.16) | -0.04 (-0.22 to 0.15) | -0.08 (-0.27 to 0.11) | -0.05 (-0.19 to 0.11) |
| Malay | 196 | -0.13 (-0.29 to 0.03) | 0.03 (-0.13 to 0.19) | -0.11 (-0.27 to 0.05) | -0.18 (-0.33 to -0.03) |
| *P* for interaction |  | 0.12 | 0.95 | 0.31 | 0.09 |
| **Head circumference (SD)**** | | |  |  |  |
| Chinese | 398 | -0.10 (-0.21 to 0.01) | -0.10 (-0.21 to 0.004) | -0.13 (-0.24 to -0.03) | -0.06 (-0.16 to 0.04) |
| Indian | 117 | -0.01 (-0.21 to 0.19) | 0.09 (-0.10 to 0.28) | -0.06 (-0.26 to 0.13) | -0.14 (-0.29 to 0.02) |
| Malay | 196 | 0.02 (-0.14 to 0.19) | -0.04 (-0.20 to 0.13) | 0.01 (-0.16 to 0.17) | 0.05 (-0.10 to 0.21) |
| *P* for interaction |  | 0.71 | 0.76 | 0.61 | 0.24 |
| **Placenta weight (SD)***** | |  |  |  |  |
| Chinese | 392 | -0.07 (-0.19 to 0.05) | -0.06 (-0.17 to 0.05) | -0.10 (-0.21 to 0.02) | -0.06 (-0.16 to 0.04) |
| Indian | 115 | -0.07 (-0.28 to 0.13) | -0.03 (-0.23 to 0.16) | -0.09 (-0.29 to 0.10) | -0.06 (-0.22 to 0.09) |
| Malay | 192 | -0.09 (-0.26 to 0.07) | -0.11 (-0.27 to 0.05) | -0.11 (-0.27 to 0.04) | -0.02 (-0.17 to 0.12) |
| *P* for interaction |  | 0.84 | 0.89 | 0.88 | 0.99 |

SD, standard deviation; CI, confidence interval.

* Measures of size at birth were estimated as gestational age adjusted standard deviation scores. Multiple linear regression models were used with adjustment for baby's sex, maternal age, ethnicity, education, parity, smoking history, height, BMI at 27 weeks gestation, coffee consumption and depression

** There were 2 women with missing information on length and head circumference

*** There were 14 women with missing information on placental weight

**Table S6. Per 1-SD Increase in Maternal Blood Pressures and Gestational Age Adjusted SD Scores of Birth Size Outcomes by Maternal BMI According to WHO Classification***

| **Maternal BMI According to WHO Classification** | **N** | **Peripheral Systolic Blood Pressure**  **(1 SD = 11.1 mmHg)** | **Peripheral Diastolic Blood Pressure**  **(1 SD = 8.3 mmHg)** | **Central Systolic Blood Pressure**  **(1 SD = 10.0 mmHg)** | **Central Pulse Pressure**  **(1 SD = 6.5 mmHg)** |
| --- | --- | --- | --- | --- | --- |
|  |  | **ß (95% CI)** | **ß (95% CI)** | **ß (95% CI)** | **ß (95% CI)** |
| **Weight (SD)** |  |  |  |  |  |
| BMI <25.0 kg/m^2^ | 329 | -0.19 (-0.31 to -0.08) | -0.09 (-0.21 to 0.01) | -0.21 (-0.32 to -0.10) | -0.18 (-0.28 to -0.08) |
| BMI 25.0-29.9 kg/m^2^ | 259 | -0.06 (-0.19 to 0.09) | -0.17 (-0.30 to -0.03) | -0.11 (-0.24 to 0.04) | 0.05 (-0.07 to 0.17) |
| BMI ≥30.0 kg/m^2^ | 125 | 0.05 (-0.18 to 0.29) | 0.24 (-0.00 to 0.48) | 0.13 (-0.10 to 0.37) | -0.06 (-0.26 to 0.14) |
| *P* for interaction |  | 0.05 | 0.01 | 0.02 | 0.009 |
| **Length (SD)**** |  |  |  |  |  |
| BMI <25.0 kg/m^2^ | 327 | -0.18 (-0.31 to -0.06) | 0.08 (-0.19 to 0.04) | -0.21 (-0.33 to -0.09) | -0.19 (-0.31 to -0.08) |
| BMI 25.0-29.9 kg/m^2^ | 259 | 0.02 (-0.12 to 0.17) | -0.15 (-0.29 to -0.01) | -0.04 (-0.18 to 0.11) | 0.11 (-0.01 to 0.23) |
| BMI ≥30.0 kg/m^2^ | 125 | -0.02 (-0.23 to 0.49) | 0.28 (0.06 to 0.49) | 0.08 (-0.14 to 0.29) | -0.16 (-0.33 to 0.02) |
| *P* for interaction |  | 0.04 | 0.005 | 0.03 | <0.001 |
| **Head circumference (SD)**** | | |  |  |  |
| BMI <25.0 kg/m^2^ | 327 | -0.15 (-0.326 to -0.03) | -0.15 (-0.26 to -0.04) | -0.18 (-0.29 to -0.06) | -0.06 (-0.17 to 0.04) |
| BMI 25.0-29.9 kg/m^2^ | 259 | -0.05 (-0.19 to 0.09) | -0.06 (-0.19 to 0.08) | -0.08 (-0.22 to 0.06) | -0.03 (-0.15 to 0.09) |
| BMI ≥30.0 kg/m^2^ | 125 | 0.14 (-0.07 to 0.36) | 0.26 (0.04 to 0.48) | 0.13 (-0.09 to 0.34) | -0.08 (-0.27 to 0.10) |
| *P* for interaction |  | 0.04 | 0.002 | 0.03 | 0.88 |
| **Placenta weight (SD)***** | |  |  |  |  |
| BMI <25.0 kg/m^2^ | 323 | -0.16 (-0.28 to -0.03) | -0.10 (-0.23 to 0.02) | -0.18 (-0.30 to -0.06) | -0.12 (-0.24 to -0.01) |
| BMI 25.0-29.9 kg/m^2^ | 256 | -0.03 (-0.17 to 0.09) | -0.08 (-0.21 to 0.05) | -0.08 (-0.21 to 0.05) | -0.01 (-0.12 to 0.10) |
| BMI ≥30.0 kg/m^2^ | 120 | 0.01 (-0.21 ti 0.24) | 0.07 (-0.17 to 0.30) | 0.04 (-0.19 to 0.26) | -0.02 (-0.21 to 0.18) |
| *P* for interaction |  | 0.09 | 0.31 | 0.07 | 0.19 |

SD, standard deviation; CI, confidence interval; BMI, body mass index; WHO, World Health Organization.

* Measures of size at birth were estimated as gestational age adjusted standard deviation scores. Multiple linear regression models were used with adjustment for baby's sex, maternal age, ethnicity, education, parity, smoking history, height, BMI at 27 weeks gestation, coffee consumption and depression.

** There were 2 women with missing information on length and head circumference.

*** There were 14 women with missing information on placental weight.
